# P09-05 Can physical activity make up for the self-care disability effects of too much sitting?

**DOI:** 10.1093/eurpub/ckac095.135

**Published:** 2022-08-29

**Authors:** Jesus del Pozo-Cruz, Borja del Pozo-Cruz, Rosa M Alfonso-Rosa

**Affiliations:** 1 Educación Física y Deporte, Universidad de Sevilla, Sevilla, Spain; 2 Australian Catholic University, Sydney, Australia; 3 Motricidad Humana y Rendimiento Deportivo, Universidad de Sevilla, Sevilla, Spain

**Keywords:** sedentary time, moderate-to-vigorous physical activity, nursing home

## Abstract

**Objectives:**

To determine whether or not, and, what extent the association between sedentary time, moderate to vogorous physical activity and self-care disability.

**Methods:**

Sedentary time was measured with accelerometers. Self-care disability was assessed with th barthel index. A multivariate regression model was used to ascertain the effects of the interaction between sedentary time and moderate to vigorous physical activity on the disability of participants. The Jhonson-Nyeman model was used to estimate the exact mvpa thresfold from which the effect of sedentary time on disability ceased to be significant.

Design: Cross-sectional

Participants: 122 (84 females, mean age 85 years old) older adults.

**Results:**

We found a significant effect of sedentary time on self care disability (p > 0.5). The results also indicated that mvpa moderates the relationship between self-care disability satatus and sedentary time. 51 min/day of mvpa would neglect the sedentary time effects on self care disability.

**Conclusions:**

The negative effects of sedentary time on self care disability can be offset with 51 min/day of mvpa.

